# High risk of acute kidney injury in Malawian trauma patients: a prospective observational cohort study

**DOI:** 10.1186/s12882-021-02564-y

**Published:** 2021-10-28

**Authors:** Erica C. Bjornstad, Zachary H. Smith, William Muronya, Charles K. Munthali, Amy K. Mottl, Stephen W. Marshall, Yvonne M. Golightly, Keisha Gibson, Anthony Charles, Emily W. Gower

**Affiliations:** 1 Department of Pediatrics, Division of Nephrology, University of Alabama at Birmingham, 1600 7th Avenue South, Lowder 516, Birmingham, AL 35233 USA; 2 Univeristy of North Carolina Project Malawi, Lilongwe, Malawi; 3 Division of Pediatric Critical Care Medicine, Stanford University School of Medicine, Stanford, USA; 4 Department of Surgery, Kamuzu Central Hospital, Lilongwe, Malawi; 5 Department of Medicine, Renal Unit, Kamuzu Central Hospital, Lilongwe, Malawi; 6 Department of Medicine, Division of Nephrology and Hypertension, University of North Carolina, Chapel Hill, NC USA; 7 University of North Carolina Injury Prevention Research Center, Chapel Hill, USA; 8 Department of Epidemiology, University of North Carolina at Chapel Hill Gillings School of Global Public Health, Chapel Hill, NC USA; 9 Department of Surgery, University of North Carolina, Chapel Hill, NC USA; 10 Malawi Surgical Initiative, Lilongwe, Malawi

**Keywords:** Trauma, Acute kidney injury, Africa

## Abstract

**Background:**

Trauma is a common cause of acute kidney injury (AKI). Yet little data exist regarding trauma-related-AKI in low-resourced settings, where the majority of deaths from AKI and trauma occur. We prospectively evaluated epidemiology of AKI in hospitalized Malawian trauma patients.

**Methods:**

AKI was defined by creatinine-only Kidney Disease Improving Global Outcomes (KDIGO) criteria. Those with AKI were followed up 3–6 months later to determine persistent kidney abnormalities. We calculated univariate statistics with Wilcoxon rank sum tests, Fisher’s exact, and chi-square tests to compare those with and without AKI. Multivariate log-risk regression modelling was used to determine risk ratios (RR) and 95% confidence intervals (CI) for AKI development.

**Results:**

Of 223 participants, 14.4% (*n* = 32) developed AKI. Most patients were young (median age 32) males (*n* = 193, 86.5%) involved in road traffic injuries (*n* = 120, 53.8%). After adjusting for confounders, those with severe anemia during their admission were 1.4 times (RR 1.4, 95% CI 1.1–1.8) more likely to develop AKI than those without. Overall mortality was 7.6% (*n* = 17), and those who developed AKI were more likely to die than those who did not (18.8% vs 5.6%, *p*-value = 0.02). Almost half of those with AKI (*n* = 32) either died (*n* = 6) or had persistent kidney dysfunction at follow-up (*n* = 8).

**Conclusion:**

In one of the few African studies on trauma-related AKI, we found a high incidence of AKI (14.4%) in Malawian trauma patients with associated poor outcomes. Given AKI’s association with increased mortality and potential ramifications on long-term morbidity, urgent attention is needed to improve AKI-related outcomes.

**Supplementary Information:**

The online version contains supplementary material available at 10.1186/s12882-021-02564-y.

## Background

Acute kidney injury (AKI) is a hidden pandemic, a devastating complication known to increase morbidity and mortality in a variety of conditions [1–3]. AKI is a sudden decline in kidney function from poor perfusion, poor oxygen delivery, toxins, direct injury, or any combination thereof. AKI is a known complication of trauma, and contributes to increased mortality risk [4–6]. Yet trauma-related AKI in low-resourced settings is not well studied. Only three studies have specifically evaluated AKI in adult trauma patients in Africa [7–9]. These studies all occurred in South Africa and found rates of AKI from 5 to 15%. However, other studies in a variety of settings (general and ICU care) worldwide have found AKI in up to 50% of admitted trauma patients, and 26% with severe AKI [10].

Trauma is a leading cause of mortality worldwide, particularly in young adults and predominantly in Africa. It is estimated that in 2013, 5 million people globally died from trauma [11]. If AKI contributes to the high mortality associated with trauma, it is imperative to understand the epidemiology further to provide evidence-based triage and prevention tools to mitigate the poor outcomes associated with trauma-related AKI. Low-resourced areas account for a disproportionate amount of the burden of both trauma and AKI [2, 3, 12]. For example, injuries cause major mortality and morbidity in Malawi; it is estimated that injuries cause 6.4% of all deaths in the nation [13]. This is approximately 1.4–4 times higher the percentage of deaths due to accidents throughout nations in the European Union [14]. Yet no study has evaluated the incidence of trauma-related AKI in Malawian adults.

We developed a prospective cohort to evaluate the incidence at a tertiary-level trauma referral center in Malawi, and to determine if there were specific risk factors that contributed to AKI development. The ethical review committees at the University of North Carolina at Chapel Hill (UNC) and Malawi’s National Health Science Research Committee approved the study.

## Methods

### Study design

We used a prospective observational cohort design to capture the incidence of AKI among admitted trauma patients and explore potential risk factors for the development of AKI and adverse clinical outcomes. Standardized questionnaires were completed by trained data clerks, and study nurses obtained all specimen samples. All methods and reporting follow STROBE guidelines.

### Setting

The study occurred at Kamuzu Central Hospital (KCH), the tertiary referral center and only trauma center for the Central Region of Malawi, which serves a population of 7.5 million people. Trauma services provided include trained surgeons, orthopedic specialists, and an intensive care unit with the ability to ventilate up to 8 patients at any given time. Laboratory services are available on site, but there are routinely electricity outages, reagent shortages, and other limitations that can make some laboratory tests that may be considered routine in other settings (i.e., serum creatinine or electrolyte tests) difficult to obtain in a timely manner. Dialysis services are available, but only for relatively stable patients as there is no ability to do continuous kidney replacement therapy, and there are only 6–8 working machines at any given time for all outpatient and inpatient services.

### Patients

All patients presenting with trauma to KCH are included in an ongoing trauma surveillance registry. For our prospective study, we included all admitted patients with an expected admission > 24 h, and this analysis focuses on the adult patients (> 18 years of age). The pediatric analysis has already been published [15]. Informed consent was obtained from all patients, or if they had altered mental status, a guardian needed to be present and informed consent obtained within 18 h of patient arrival. Patients were excluded if they did not speak English or Chichewa (the Malawian national language) or if their trauma occurred > 5 days prior to arrival. Subjects were enrolled from June–October 2018.

### Sample size

To estimate the AKI incidence in adult trauma patients with +/− 5% precision, we calculated that we would need to enroll 218 subjects. This assumes an AKI incidence in the range of 15–20%, which is consistent with prior research [16]. We elected to enroll 240 to account for potential attrition and other complications.

### Outcomes

The primary outcome of interest was the development of AKI, defined by serum creatinine changes and thresholds according to the Kidney Disease Improving Global Outcomes (KDIGO) criteria [17]. As no one had baseline creatinine values available, these were estimated using the Modification of Diet in Renal Disease (MDRD-4) equation as recommended by KDIGO criteria for adults excluding the ethnicity factor and assumed an eGFR of 75 ml/min/1.73m^2^. Newer equations are used for estimating eGFR in stable adult patients, and we did a sensitivity analysis to assess if changing the equation for baseline creatine estimation altered our findings. We did not include urine output measurements in the definition of AKI as they are not reliable in this setting.

We obtained serum creatinine values on admission (within 18 h of arrival) and 48–72 h later. Serum creatinine values were obtained on fresh serum with Roche Cobas C311 analyzers using the Jaffe analytic method at the UNC Project Malawi Laboratory, an internationally accredited research facility on-site at KCH. Additional outcomes included hospital mortality, duration of hospitalization, and receipt of dialysis.

UNC laboratory facilities are not open every day; they are closed Saturday midday to Monday morning. Therefore, we allowed admission samples to be obtained within 18 h of presentation to minimize bias in case weekend admissions differed systematically from weekday admissions.

### Variables

As this was an exploratory analysis to determine potential risk factors of AKI, a variety of readily available data and pertinent clinical information were sought and included. Patients/caregivers were asked a standard survey about demographics, injury characteristics, socioeconomic status (i.e., education level, flooring type, crowding), and known chronic conditions. The Kampala Trauma Score (a validated score for injury severity in low-resourced settings) [18] was determined for patients who had all components documented in their medical file.

As outpatient primary care access is minimal in Malawi, we also obtained laboratory tests for commonly implicated conditions in kidney dysfunction. All subjects had serum hemoglobin assessed with point-of-care testing (HemoCue Hb 201+ analyzer) to determine anemia as defined by the World Health Organization: hemoglobin< 12 g/dL for women and < 13 g/dL for men. In addition, we defined severe anemia as hemoglobin< 10 g/dL. All subjects were assessed for sickle cell trait or disease by hemoglobin electrophoresis and for malaria by blood smears at UNC Laboratory. Human immunodeficiency virus serostatus was determined by standard hospital protocols through documentation or self-report by patient.

### Follow-up

We attempted to contact all subjects identified as having AKI 3–6 months after hospital discharge. For logistical reasons, the follow-up visits were coordinated to occur on 1 day in January 2019. At follow-up, we obtained serum creatinine, urinalysis for proteinuria, and blood pressure measurements for hypertension.

### Statistical analysis

Sociodemographic, injury-related, and clinical characteristics are presented with descriptive statistics. Univariate statistics between those who did and did not develop AKI were evaluated with chi-square (Fisher’s exact for small numbers) and Wilcoxon rank sum tests for categorical and continuous variables, respectively. For variables with *p*-value ≤0.1 in univariate analysis, we conducted multivariate log-risk regression modelling to determine adjusted risk ratios for the development of AKI. Gender was automatically included in the model since it is assumed to be strongly associated with a variety of trauma-related factors and potential association with AKI.

We also conducted a sensitivity analysis of the AKI incidence depending on the different estimations of baseline creatinine. KDIGO recommends using MDRD-4 to estimate a baseline creatinine when it is not known in adult populations, but some literature suggests that a newer equation (CKD-EPI) better estimates the glomerular filtration rate (eGFR) in Black African adults [19–21]. We compared how both the MDRD-4 and CKD-EPI equations for estimating the baseline creatinine altered the incidence of AKI in this population. We used the lowest creatinine method during the hospitalization as a gold standard comparison for AKI incidence as this value has no assumptions, and in this setting, there is little that would falsely lower the value. To make the comparisons equal, the sensitivity analysis is limited to only those with 2 serum creatinine measurements.

Data were double-entered into REDCap (hosted at UNC) electronic data capture tools from paper surveys [22]. All statistical analyses were conducted in SAS, version 9.4 (SAS Institute, Inc., Cary, North Carolina).

## Results

### Incidence

In this cohort of 223 Malawian trauma patients, 14.4% (*n* = 32) developed AKI within the first 3 days of hospitalization, based on the creatinine-only KDIGO definition. The majority (62.5%) had stage 1 AKI (*n* = 20), but 7 (21.9%) developed stage 2 and 5 (15.6%) developed stage 3 AKI. Sensitivity analyses showed AKI incidences only varied slightly when different methods were used for estimating a baseline creatinine value (supplementary Table 1).

### Demographics

The patients were young adults on average, with a median age of 32 years and a minority were female (13.5%) (Table 1). There were no differences in AKI development by age, tribal association, or district where injury occurred. In univariate analyses, those with a higher level of education had a higher risk of AKI development, 7.8 ± 3.6 years versus 6.4 ± 3.8 years, *p*-value = 0.04 (Table 2). Among other indicators of socioeconomic status (i.e., crowding, type of flooring) only a deceased mother was slightly associated with an increased risk of AKI development (50% versus 36.6%, *p*-value = 0.1) (Table 2). We evaluated demographic and injury characteristics between those enrolled versus not enrolled and found no significant differences in age, gender, or mechanisms of trauma between the groups.

**Table 1:** Demographics and outcomes of adult trauma patients admitted to the hospital by development of AKI in Malawi

	Total	AKI	No AKI	*p*-values
Characteristics and Outcomes	223	32 (14.4)	191 (85.7)	
**Age, years (median, IQR)**^a^	32 (25, 42)	34 (27.5, 46)	32 (25, 42)	0.3
**Gender (female)**	30 (13.5)	4 (12.5)	26 (13.6)	1.0
**Tribe**^a^				0.2
Chewa	138 (61.9)	20 (62.5)	118 (61.8)	
Lomwe	17 (7.6)	1 (3.1)	16 (8.4)	
Ngoni	35 (15.7)	3 (9.4)	32 (16.8)	
Other	31 (13.9)	8 (25.0)	23 (12.0)	
**District of injury location**^a^				0.6
Lilongwe District	155 (69.5)	21 (65.6)	134 (70.2)	
Other District	67 (30.0)	11 (34.4)	56 (29.3)	
**Comorbidities**				
Self-report of Chronic Conditions				
Seizures	10 (4.5)	4 (12.5)	6 (3.1)	0.04
Hypertension	6 (2.7)	1 (3.1)	5 (2.6)	1.0
Other	6 (2.7)	2 (6.3)	4 (2.1)	0.2
HIV^a^	13 (5.8)	0 (0)	13 (6.8)	0.2
Anemia (females< 12 g/dL, males< 13 g/dL)	151 (67.7)	30 (93.8)	121 (63.4)	0.0004
Severe Anemia (Hg < 10 g/dL)	60 (26.9)	17 (53.1)	43 (22.5)	0.0003
Malaria^a^	10 (4.5)	0 (0)	10 (5.2)	0.4
Sickle Cell Disease^a^				0.2
Sickle Cell Trait	20 (9.0)	5 (15.6)	15 (7.9)	
Normal	193 (86.5)	25 (78.1)	168 (88.0)	
**Mortality**^**a**^	17 (7.6)	6 (18.8)	11 (5.6)	0.02
**Length of hospitalization, days, median (IQR)**^**b**^	10 (5, 29)	14 (7.5, 47.5)	9 (5, 28)	0.1

All data presented as n(column %) except where specified

^a^ Missing variables for age (*n* = 2), tribe (*n* = 2), injury location (*n* = 1), mortality (*n* = 14), time of day (*n* = 1), HIV status (*n* = 159, 71.3%), malaria (*n* = 2), sickle cell status (*n* = 10)

^b^Among survivors, excluding 17 who died and 15 with unknown length of stay or left against medical advice

**Table 2:** Socioeconomic status variables among adult trauma patients by development of AKI in Malawi

	Total	AKI	No AKI	*p*-values
Socioeconomic Status Variables	223	32 (14.4)	191 (85.7)	
**Education (mean, std)**^**a**^				
Patient	6.6 (3.8)	7.8 (3.6)	6.4 (3.8)	0.04
Patient’s Mother	3.8 (4.0)	4.8 (4.7)	3.7 (3.9)	0.4
Patient’s Father	4.7 (4.4)	4.9 (4.6)	4.7 (4.3)	0.9
**Patient’s Level of Education**^**a**^				0.1
Secondary or more (9 or more years)	64 (28.7)	14 (43.8)	50 (26.2)	
Some primary (1–8 years)	132 (59.2)	16 (50.0)	116 (60.7)	
No formal education	16 (7.2)	1 (3.1)	15 (7.9)	
**Crowding Factor (mean, std)**^**b**^	1.5 (0.8)	1.4 (0.7)	1.5 (0.8)	0.5
**Type of Roof**^**a**^				0.2
Homeless	1 (0.4)	0 (0)	1 (0.5)	
Thatch	73 (32.7)	11 (34.4)	62 (32.5)	
Tin/Iron	141 (63.2)	20 (62.5)	121 (63.4)	
Tile	7 (3.1)	0 (0)	7 (3.7)	
**Type of Floor**				0.4
Dirt/Mud	103 (46.2)	12 (37.5)	91 (47.6)	
Concrete/Cement	119 (53.4)	20 (62.5)	99 (51.8)	
Tile	1 (0.5)	0 (0)	1 (0.5)	
**Patient’s Parent(s) Deceased**^**c**^				
Mother	86 (38.6)	16 (50.0)	70 (36.6)	0.1
Father	115 (51.6)	16 (50.0)	99 (51.8)	0.9
Both	69 (30.9)	12 (37.5)	57 (29.8)	0.3

All expressed as n(column %) except where specified

^a^ Missing variables for patient’s education (*n* = 11), mother’s education (*n* = 102), father’s education (*n* = 100), type of roof material (*n* = 1), whether or not parents’ deceased (*n* = 3)

^b^ Crowding factor = number of permanent residents in a home divided by number of contiguous rooms in a housing unit

^c^ exclusive (ie, if both parents deceased, individual also included in row of mother and father deceased)

Individuals reporting history of seizures had a higher likelihood of AKI development (*p*-value = 0.04), but no other self-reported chronic conditions were associated with AKI development (Table 1). We also found that anemia, and particularly severe anemia (hemoglobin< 10 g/dL), was associated with increased risk of AKI (*p*-value< 0.001). There was insufficient reporting of HIV status to determine its influence on AKI development. Laboratory testing for malaria (endemic in this region) and sickle cell status did not reveal differences in AKI risk (Table 1).

### Injury-related and Nephrotoxin exposures

Only slight differences were seen in the injury-related exposures and AKI development (Table 3). Those with multiple injuries (*p*-value = 0.1), trunk injuries (*p*-value = 0.1), burn-related injuries (*p*-value = 0.09), and injuries occurring within 24 h of hospital presentation (*p*-value = 0.1) were slightly associated with an increased risk of AKI in univariate analyses. There were no differences in AKI development among potential concomitant nephrotoxic exposures (medications, herbal remedies, drinking water source, CT scan) (Table 3).

**Table 3:** Trauma-related and nephrotoxic exposure-related factors amongst admitted adult trauma patients in Malawi by development of AKI

Potential Trauma-related and Nephrotoxic-related Factors	Total	AKI	No AKI	*p*-values
	223	32 (14.4)	191 (85.7)	
**Time of Presentation to Hospital**				
Day of the week (weekend, Saturday-Sunday)	42 (18.8)	6 (18.8)	36 (18.9)	1.0
Time of day (Daytime: 08:00–16:00)^a^	70 (31.4)	12 (37.5)	58 (30.4)	0.4
**Type of Trauma**^**a**^				
Road Traffic Injury	120 (53.8)	16 (50.0)	104 (54.5)	0.6
Assault	55 (24.7)	8 (25.0)	47 (24.6)	1.0
Fall	22 (9.9)	3 (9.4)	19 (9.9)	1.0
Collapsed Structure	13 (5.8)	2 (6.3)	11 (5.8)	1.0
Burn	8 (3.6)	3 (9.4)	5 (2.6)	0.09
Other	3 (1.3)	0 (0)	3 (1.6)	1.0
**Kampala Trauma Score (mean, std)**^**a**^	14.5 (1.1)	14.1 (1.7)	14.5 (1.1)	0.6
**Primary Location of Trauma**^**a**^				1.0
Head/Neck	66 (29.6)	9 (28.1)	57 (29.8)	
Face	30 (13.5)	5 (15.6)	25 (13.1)	
Trunk	34 (15.2)	5 (15.6)	29 (15.2)	
Extremity	91 (40.8)	12 (37.5)	79 (41.4)	
**Multiple injuries vs single injuries**^**a**^	128 (57.4)	22 (68.8)	106 (55.5)	0.1
**All Trauma Locations**^**b**^				
Head/Neck	82 (36.8)	10 (31.3)	72 (37.7)	0.6
Face	56 (25.1)	8 (25.0)	48 (25.1)	0.9
Trunk	62 (27.8)	12 (37.5)	50 (26.2)	0.1
Extremity	139 (62.3)	21 (65.6)	118 (61.8)	0.5
**Injury occurred < 24 h prior to arrival**^**a**^	184 (82.5)	30 (93.8)	154 (80.6)	0.1
**Drinking water source**^**a**^				0.4
River/lake	15 (6.7)	2 (6.3)	13 (6.8)	
Community pipe/Bore Hole	123 (55.2)	14 (43.8)	109 (57.1)	
Piped (exterior)	51 (22.9)	6 (18.8)	45 (23.6)	
Piped (interior)	28 (12.6)	6 (18.8)	22 (11.5)	
other	2 (0.9)	1 (3.1)	1 (0.5)	
**Medications taken in preceding 7 days**	70 (31.4)	9 (28.1)	61 (31.9)	0.7
**Herbal remedies taken in previous 7 days**	33 (14.8)	4 (12.5)	29 (15.2)	1.0
**CT scan during admission**^**a**^	5 (2.2)	1 (3.1)	4 (2.1)	0.5

All expressed as N and column percent except where specified

Categories are mutually exclusive except where specified

*AKI* Acute kidney injury. KDIGO criteria used to define AKI and MDRD-4 equation estimated baseline creatinine

^a^ Missing variables for time of presentation (*n* = 1), type of trauma (*n* = 2), Kampala Trauma Severity Score (*n* = 129, majority missing due to a lack of documented respiratory rate, 105 of 129), primary injury location (*n* = 2), single vs multiple injuries (*n* = 2), injury timing (*n* = 3), drinking water source (*n* = 4), CT scan (*n* = 15)

^b^Multiple categories allowed. Missing data on 1 participant

### Independent risk factors for AKI development

**Fig. 1:**
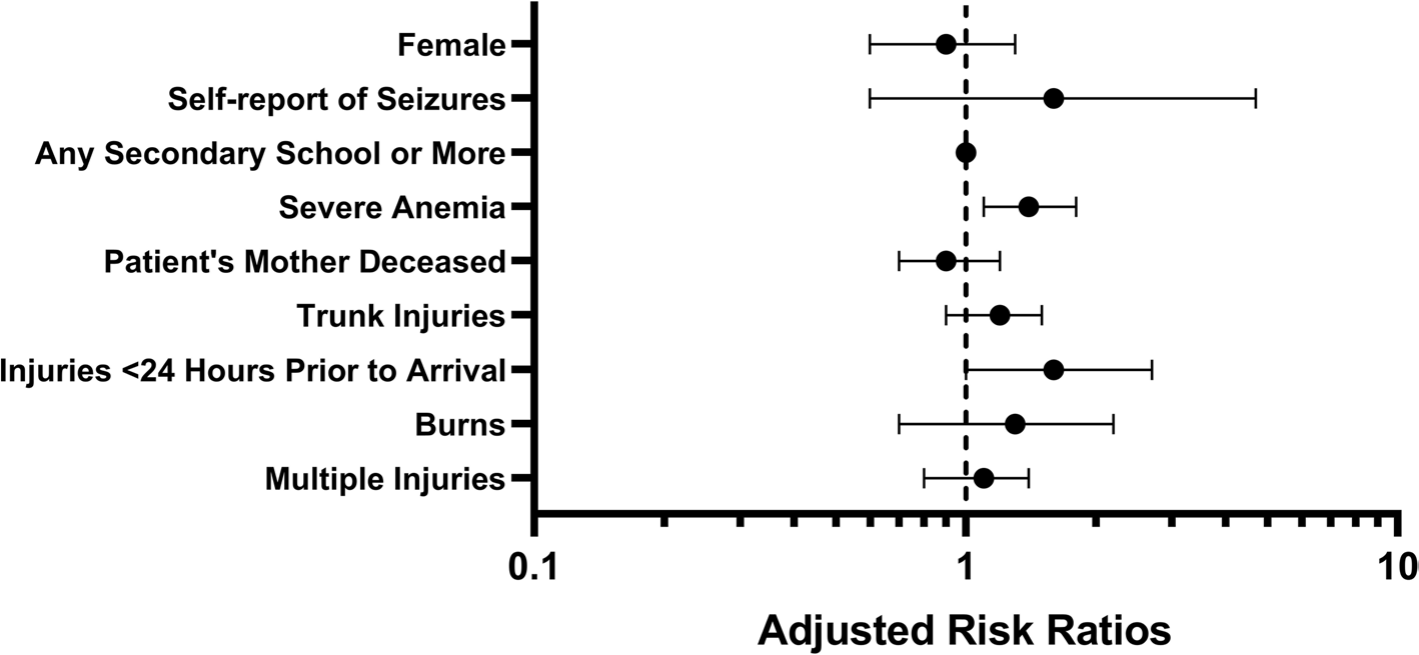
Adjusted Risk Ratios for AKI Development in Malawian Trauma Patients. Adjusted risk ratios for AKI development are presented on a logarithmic scale with 95% confidence intervals. Variables are compared to the absence of stated variable, except as follows: females versus males, any secondary school versus less than secondary school as highest level of education attainment, and multiple injuries versus single injuries

In multivariable regression analyses, severe anemia was the largest independent risk factor for the development of AKI in this cohort (Fig. 1), with a 1.4-fold increased risk (95% CI 1.1, 1.8, *p*-value< 0.01) of developing AKI for those who had severe anemia. Though several other variables were significant in univariate analyses, these did not hold when controlling for other factors. The only other variable that approached statistical significance was time between injury and presentation to the hospital. There was a tendency towards an increased risk of developing AKI for those who presented sooner than 24 h compared to those who presented later (RR = 1.6, 95% CI 1.0, 2.7, *p*-value = 0.06).

### Clinical outcomes

Among our cohort, 7.6% (*n* = 17) of hospitalized trauma patients died during hospitalization (Table 1). Those who developed AKI were more likely to die than those who did not (18.8% versus 5.6%, *p*-value = 0.02). In addition, among those who survived to hospital discharge (*n* = 191), those who developed AKI had a longer median hospitalization compared to those who did not develop AKI (14 versus 9 days), though the difference was not statistically significant (Table 1). No patient underwent dialysis; though this should be interpreted with caution as dialysis services are limited to stable patients.

Almost half of all patients who developed AKI either died or had long-term kidney complications (14 of 32). However, we were only able to reach 16 of the 26 who were discharged alive. Six of 32 patients died while hospitalized. Of the remaining 26, 10 returned for follow-up within 3–6 months after hospital discharge and 8 had persistent kidney dysfunction (isolated abnormal eGFR (*n* = 5) and remaining *n* = 3 had abnormal eGFR, hypertension and proteinuria).

## Discussion

In this first evaluation of trauma-related AKI in hospitalized Malawian patients, we found a high AKI incidence, 14.4%, within the first 3 days of admission. We found similar AKI rates to critically-ill hospitalized trauma patients in South Africa (15%), though we included both non-critical and critically ill patients [9]. However, a similar retrospective study of 6367 critically ill and non-critically ill admitted South African adult trauma patients reported a lower incidence of AKI (5.8%) [8]. That study, however, relied on retrospective data and 30% did not have an admission serum creatinine, which may have biased their results. AKI is not readily detected clinically and relies on laboratory tests for confirmation. In Malawi and other lower-income settings, serum creatinine is not routinely assessed in trauma patients, and our study highlights similar to the South African studies that this needs further assessment. Prospective evaluations are essential given the frequency of absent creatinine tests in general trauma patients in low-resourced settings.

As one of the first studies on AKI in trauma patients in a low-income setting, this was exploratory to identify potential risk factors for AKI development. We found that severe anemia (hemoglobin < 10 g/dL) was a significant risk factor for developing AKI. There are two potential mechanisms by which severe anemia could lead to AKI. Poor oxygen delivery due to decreased hemoglobin can lead to proximal tubule necrosis, also known as acute tubular necrosis, and subsequent AKI. Significant blood loss from trauma itself can lead to overall decreased kidney perfusion, which also subsequently will lead to kidney ischemia and AKI, and this significant blood loss can be reflected as a low hemoglobin level as well. Since the 1950s and more recent observational studies have also found that anemia is an acute predictor of AKI [23–26]. We confirmed this observation in a low-resourced setting as well. However, simply providing blood transfusions to those with severe anemia may not be a straightforward solution as has been seen in several observational studies surrounding perioperative AKI development, anemia, and blood transfusions [27–29]. Blood transfusions themselves at certain hemoglobin levels could be detrimental as discussed in this review, potentially due to decreased oxygen carrying capacity of pRBCs, or perhaps as a surrogate for more severe disease or extensive surgery or trauma [30]. There is likely a complex interplay between anemia and its therapy of blood transfusions, and future interventional studies should consider optimal transfusion thresholds in trauma patients in low-resourced settings to potentially improve AKI- and trauma-related outcomes.

We found that AKI was associated with a much higher risk of mortality (18.8%) compared to those without AKI (5.6%) among hospitalized trauma patients. This finding is similar to other studies in South Africa and high-income settings [8, 31–33]. This increased risk of mortality was also seen in our previous analysis of pediatric trauma patients [15]. As Malawi has one of the highest rates of road traffic injuries in the world [34, 35], confirming that trauma leads to high AKI rates in low-income settings is important as this may be an intervenable target to improve trauma-related mortality.

In other conditions, AKI is known to increase the risk of long-term morbidity as well as mortality [36–40]. In addition to increased mortality in trauma patients, we also found that of those who survived hospital discharge with AKI a majority may have persistent kidney abnormalities 3–6 months later. Half of those who developed AKI had poor outcomes (6 died, 8 had persistent kidney dysfunction, hypertension and/or proteinuria); though we were unable to contact 16 of the 26 patients (61.5%) with AKI who were discharged alive. Therefore, this may be an underestimation of poor outcomes associated with trauma-related AKI. In a recent study from South Africa, it was similarly found that critically ill adults, who developed AKI and discharged alive, 13% had prolonged kidney dysfunction at follow-up [4]. That study only looked at abnormal eGFR, whereas our study evaluated abnormal eGFR, hypertension and proteinuria, which are all associated with poor kidney outcomes. Our study adds to the evidence that long-term AKI follow-up studies are desperately needed in Africa. The poor kidney outcomes associated with AKI may have implications for the rising burden in low-resourced settings of chronic kidney disease and need for chronic dialysis, which can be quite limited or absent in these settings, and AKI can be postulated to have easier management solutions compared to chronic kidney failure.

### Limitations

Our study is not without limitations as we were only able to obtain at most 2 serum creatinine values per patient to identify an episode of AKI and other studies tend to evaluate 5–7 daily measurements. This was not feasible in this setting, but as our study was prospective and obtained creatinine values on all admitted trauma patients adds to our understanding of AKI in this high-risk population. The study was designed to accurately assess the incidence of AKI, not to evaluate all potential risk factors for AKI. Though we found that anemia remained a significant risk factor for AKI, we cannot draw definitive conclusions about other risk factors whose *p*-values are larger, as there may not have been sufficient participants for fully evaluating those risk factors (e.g., specific trauma types). This analysis is part of a larger study that also evaluated novel biomarkers for AKI detection, and as such participants/caregivers were required to give consent prior to participation, which meant that those who arrived and died shortly after arrival or with altered mental status and no caregiver present were excluded. In addition, those with severe injuries who died on the scene or in transport could not be captured. Therefore, it is likely that our estimate of AKI is an underestimation of the true overall incidence of AKI in adult trauma patients in Malawi.

## Conclusion

To our knowledge, this is the first study in a low-income setting in sub-Saharan Africa that prospectively evaluated AKI in trauma patients. We found a high incidence of early AKI (14.4%) associated with an increased risk of mortality and potentially long-term kidney dysfunction among survivors with AKI. Half of those who developed AKI had poor outcomes. Research in higher-income settings suggests that earlier recognition and prevention of AKI can improve outcomes [41, 42]. With the high burden of trauma-related AKI in this population, this should be explored further to evaluate if preventing and earlier treatment of AKI can improve trauma-related outcomes. In particular, our finding of severe anemia being an independent risk factor for AKI development is an intriguing finding that could potentially be an intervention target to improve outcomes. Given the increased recognition of chronic kidney disease burden in sub-Saharan Africa [43, 44], further research should explore how AKI may be contributing to that burden. This may be an area of intervention further upstream to delay or prevent the need for chronic dialysis and transplant which can be prohibitively expensive, or simply not available, in many areas in sub-Saharan Africa. At a minimum, hospitalized trauma patients need to be screened for AKI development given its increased association with poor outcomes.

## Supplementary Information

**Additional file 1: Supplementary Table 1.** Incidence of AKI and Outcomes by Definition of Baseline Creatinine Estimation Method.

## Data Availability

The datasets used and analysed during the current study are available from the corresponding author on reasonable request.
