# Supplementary material for: High risk of acute kidney injury in Malawian trauma patients: a prospective observational cohort study
Source: BMC Nephrol. 2021 Oct 28;22:354. doi: 10.1186/s12882-021-02564-y (PMC8552973; doi:10.1186/s12882-021-02564-y)
Supplement: Supplementary file 1 — Additional file 1: Supplementary Table 1. Incidence of AKI and Outcomes by Definition of Baseline Creatinine Estimation Method. [file 12882_2021_2564_MOESM1_ESM.docx]

**Supplementary Table 1. Incidence of AKI and Outcomes by Definition of Baseline Creatinine Estimation Method.**

| **Baseline Creatinine Estimation Method** | **Total AKI** | **AKI Stage 2-3** | **Mortality among those with AKI** | **Long-term Renal Dysfunction*** |
| --- | --- | --- | --- | --- |
|  | *N=165* | *N=165* | *N=13* | *N=9* |
| **Lowest Creatinine** | 27 (16.4) | 8 (4.9%) | 6 (46.2%) | 5 (55.6%) (uses CKD-EPI to define eGFR) |
|  |  |  |  | 7 (77.8%) (uses MDRD to define eGFR) |
| **CKD-EPI** | 26 (15.8) | 4 (2.4%) | 5 (38.5%) | 5 of 9 (55.6%) |
| **MDRD-4** | 29 (17.6) | 11 (6.7%) | 5 (38.5%) | 7 of 9 (77.8%) |
| Restricted to those with at least 2 creatinine values  All presented as n(column %). | | | | |
| * Among those with AKI by given definition (baseline creatinine estimation), those who had persistent long-term renal dysfunction at follow-up (defined as proteinuria 1+ or greater, hypertension, or eGFR <90ml/min/1.73m2 as determined by given equation). | | | | |
